# Level of physical activity, well-being, stress and self-rated health in persons with migraine and co-existing tension-type headache and neck pain

**DOI:** 10.1186/s10194-017-0753-y

**Published:** 2017-04-18

**Authors:** Lotte Skytte Krøll, Catharina Sjödahl Hammarlund, Maria Lurenda Westergaard, Trine Nielsen, Louise Bönsdorff Sloth, Rigmor Højland Jensen, Gunvor Gard

**Affiliations:** 1 Department of Health Sciences, Lund University, P.O. Box 157, 221 00 Lund, Sweden; 2 Danish Headache Centre, Department of Neurology, Rigshospitalet-Glostrup, University of Copenhagen, Nordre Ringvej 69, 2600 Glostrup, Denmark

**Keywords:** Migraine, Neck pain, Physical activity, Psychological well-being, Tension-type headache, Self-rated health, Stress

## Abstract

**Background:**

The prevalence of migraine with co-existing tension-type headache and neck pain is high in the general population. However, there is very little literature on the characteristics of these combined conditions. The aim of this study was to investigate a) the prevalence of migraine with co-existing tension-type headache and neck pain in a clinic-based sample, b) the level of physical activity, psychological well-being, perceived stress and self-rated health in persons with migraine and co-existing tension-type headache and neck pain compared to healthy controls, c) the perceived ability of persons with migraine and co-existing tension-type headache and neck pain to perform physical activity, and d) which among the three conditions (migraine, tension-type headache or neck pain) is rated as the most burdensome condition.

**Methods:**

The study was conducted at a tertiary referral specialised headache centre where questionnaires on physical activity, psychological well-being, perceived stress and self-rated health were completed by 148 persons with migraine and 100 healthy controls matched by sex and average age. Semi-structured interviews were conducted to assess characteristics of migraine, tension-type headache and neck pain.

**Results:**

Out of 148 persons with migraine, 100 (67%) suffered from co-existing tension-type headache and neck pain. Only 11% suffered from migraine only. Persons with migraine and co-existing tension-type headache and neck pain had lower level of physical activity and psychological well-being, higher level of perceived stress and poorer self-rated health compared to healthy controls. They reported reduced ability to perform physical activity owing to migraine (high degree), tension-type headache (moderate degree) and neck pain (low degree). The most burdensome condition was migraine, followed by tension-type headache and neck pain.

**Conclusions:**

Migraine with co-existing tension-type headache and neck pain was highly prevalent in a clinic-based sample. Persons with migraine and co-existing tension-type headache and neck pain may require more individually tailored interventions to increase the level of physical activity, and to improve psychological well-being, perceived stress and self-rated health.

## Background

The prevalence of migraine in Europe is 15% [1], and persons with migraine often suffer from co-morbidities such as tension-type headache (TTH) and neck pain (NP). In a population study, 94% of persons with migraine reported co-existing TTH [2], and NP was found among 89.3% of persons with migraine with co-existing TTH [3]. The prognosis of migraine may worsen with co-existing TTH [4], and NP has been found to be a predictor of increased disability in persons with migraine [5].

This study focuses on persons with migraine and co-existing TTH and NP. There is very little literature on the characteristics of persons with migraine and co-existing TTH and NP. There are, to our knowledge, no studies that describe their symptomatology, physical activity, psychological well-being, perceived stress or self-rated health. These characterizations are important when considering potential treatment strategies.

It is not known whether specific nonpharmacological treatments could be particularly helpful for persons with migraine and co-existing TTH and NP. Lifestyle modifications like exercise, relaxation and biofeedback are recommended nonpharmacological strategies for persons with migraine [6–9]. These modalities may also be effective in treating the co-existing TTH and NP [10–13].

The aim of this study was to investigate a) the prevalence of migraine and co-existing TTH and NP in a clinic-based sample, b) the level of physical activity, psychological well-being, perceived stress and self-rated health in persons with migraine and co-existing TTH and NP compared to healthy controls, c) the perceived ability of persons with migraine and co-existing TTH and NP to perform physical activity, and d) which among the three conditions (migraine, TTH or NP) is rated as the most burdensome condition.

## Methods

### Participants

**Fig. 1:**
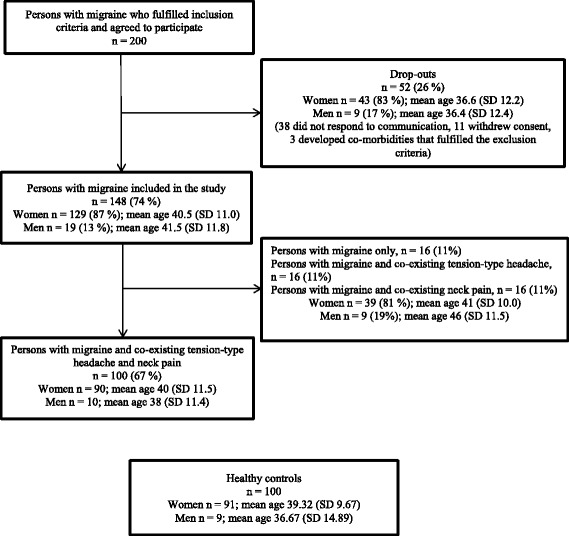
Flow chart of the inclusion procedure of a clinic-based sample of persons with migraine

Two hundred persons with migraine aged 18-65, screened for exclusion criteria and who initially accepted participation were consecutively recruited from a tertiary referral headache centre between February 2014 and March 2015 148 (74%) returned the questionnaires and were included in the study (Fig. 1).

Exclusion criteria were: whiplash injury, significant neck trauma (defined as a history of trauma to the neck, fracture, distortion and violent attack which have caused the current NP), post traumatic headache, medication-overuse headache, cluster headache, trigeminal neuralgia, pregnancy and/or breastfeeding, severe physical and/or mental diseases, abuse of alcohol or drugs and inability to speak or understand Danish.

Between June 2014 and October 2015, 100 headache-free and healthy controls were recruited among hospital staff by modified snowball sampling and advertisement. Exclusion criteria were abuse of alcohol or drugs, inability to speak and understand Danish, regular intake of analgesics, NP and TTH frequency ≥1 day per month.

The healthy controls were matched to the included persons with migraine by the average value of age and the percentage distribution of sex.

### Procedure

The participants with migraine (*n* = 148) and healthy controls (*n* = 100) completed three questionnaires which were validated for the target population in a previous study [14]; (a) The International Physical Activity Questionnaire (IPAQ short form) [15], (b) the 5-item World Health Organization Well-Being Index (WHO-5) [16] and (c) Impact of Migraine, Tension-Type Headache and Neck Pain (Impact of Migraine , TTH and NP) [14]. A maximum of three reminders were sent out.

### Interview

#### Migraine, TTH and NP

The participants filled out a 4-week diagnostic headache diary before the first visit in the headache centre. Based on data from the diary and a neurological examination the neurologist ascertained the diagnosis of migraine. They were then interviewed using a modified semi-structured headache interview [17, 18] which was supplemented with questions regarding NP. NP was defined as pain located to the anatomic region of the neck with or without radiation to the head, trunk, and upper limbs grade I-II, based on the classification system developed by the Neck Pain Task Force [19], which have set a conceptual model for the onset, course, and care of NP.

To distinguish NP from NP associated with the migraine attack itself and NP outside the migraine attack the participants were asked: “*Do you have NP associated with the migraine attack?” “Do you have NP associated with TTH?” “Do you have NP without headache?”*

The screening for inclusion and exclusion criteria and all interviews were done by specially trained medical students (TN, LBS) and the primary investigator (LSK). The semi-structured headache interview [17] was originally based on International Classification of Headache Disorders (ICHD) criteria from 1988 [20], but the interview guide provided enough information to classify migraine and TTH according to the ICHD-3 beta criteria [21]. Based on the interview, the one-year prevalence and average days of migraine, TTH and NP per month were determined.

### Questionnaires

#### IPAQ short form

The level of physical activity was measured using the IPAQ short form [14, 15]. IPAQ measures time and energy expenditure in the past 7 days. Participants were classified as having low, moderate or high levels of physical activity based on the standard algorithm [22]. The IPAQ protocol considers performed physical activity and their corresponding metabolic equivalent task (MET). One MET is equivalent to energy expenditure while resting. Participants were excluded from the analyses if they answered “don’t know/insecure” in any of the categories of walking, moderate or vigorous physical activity, and if time spent on any of the categories were unreasonably high.

#### WHO-5

Psychological well-being was measured by WHO-5 [16], which is based on five items with a two-week recall period. Responses were rated on six-point Likert categories (0 to 5). The scores were summed (maximum score 25) and then multiplied by 4 (range 0 to 100 with lower scores indicate lower well-being). The cut-off level of ≤ 50 was used to indicate poor psychological well-being, which may indicate stress or depression [23].

#### Impact of Migraine, TTH and NP questionnaire

The Impact of Migraine, TTH and NP questionnaire consists of 79 items covering pain, triggers, psychosocial, socioeconomic and work related aspects, based on a four-week recall period. The questionnaire has previously been tested for face and content validity [14]. The following items were analysed in this study: perceived stress, self-rated health, the perceived ability to perform physical activity and rating of the most burdensome condition. Perceived stress and perceived ability to perform physical activity were rated on an 11-point numeric rating scale (NRS-11) with the end points 0 = no impact and 10 = most imaginable impact as applied in previous studies [24, 25].

Perceived stress was assessed by a reliable and valid item from the QPS Nordic Questionnaire [26]: “*Stress means the situation when a person feels tense, restless, nervous, or anxious, or is unable to sleep at night because his or her mind is troubled all the time. In the past four weeks, did you feel that kind of stress these days?”* Responses ranged from 0 (not at all) to 10 (very high degree). To be able to analyse the different levels of perceived stress, we coded scores 0-3 as low, scores 4–6 as moderate and 7–10 as high.

Self-rated health was assessed by one item, originally derived from SF 36 [27]: *“In general, how would you rate your current health?”* The response categories were: 1) very poor, 2) poor, 3) fair, 4) good or 5) very good. The variable was changed to a binary categorical variable “poor self-rated health” as applied in previous studies [3, 28]. Scores 1–3 were coded as poor self-rated health.

Perceived ability to perform physical activity was assessed by three items: *“In the past four weeks, when having migraine/TTH/NP, how much has your ability to perform physical activity (physical activity or strength training) been reduced?”* Responses ranged from 0 (not reduced) to 10 (reduced to a very high degree).

Rating of the most burdensome condition was assessed by one item: *“In the past four weeks, to what extent does your migraine, TTH and NP generally affect you? If you consider all three conditions as adding up to 100%, to what extent does each of these conditions affect you?”*

### Statistical analysis

Data on demography, lifestyle and clinical characteristics of the participants were presented with descriptive statistics.

In comparing migraine and co-existing TTH and NP with the control group, Independent-samples t-test was used to test for differences in age as age was tested as normally distributed by the Shapiro-Wilk test. Chi-square test was used to test for difference in sex, educational attainment, physical activity, psychological well-being, stress and self-rated health.

A significant difference in educational attainment between migraine and co-existing TTH and NP and healthy controls was observed. Low educational level is associated with reduced health and low level of physical activity [29], therefore, separate binary logistic regressions were performed with migraine and co-existing TTH and NP as the outcome variable and the following as covariates: Physical activity (low, moderate and high), psychological well-being (score ≤ 50, yes or no), perceived stress (low, moderate and high), and poor self-rated health (yes or no). These analyses were controlled for educational attainment. Results from the logistic regression were presented as odds ratios (OR) with 95% confidence intervals (CI).

All statistical analyses were performed using IBM SPSS version 22. Statistical significance was assumed when *p* < 0.05.

## Results

The 148 persons with migraine included in the study were comprised of 106 (72%) who had migraine without aura, 8 (5%) who had migraine with aura, and 34 (23%) who had both migraines with and without aura. Among them, 100 (67%) suffered from co-existing TTH and NP. Only 11% suffered from migraine only (*n* = 16), 11% suffered from migraine and co-existing TTH (*n* = 16), and 11% had migraine and co-existing NP (*n =* 16).

Data on the participants’ demographic characteristics, educational attainment, physical activity, psychological well-being, perceived stress, self-rated health, headache and NP are summarized in Table 1. There were no differences in sex and age, but educational attainment was significantly different, between migraine and co-existing TTH and NP and healthy controls.

**Table 1:** Health-related variables, headache and neck pain frequencies in persons with co-existing M, TTH and NP

	All *N* = 148	M-TTH-NP *N* = 100	Healthy controls *N* = 100	*p* value M-TTH-NP healthy controls
Age, mean (SD)	41 (11.1)	40 (11.4)	39 (10.2)	0.49
Sex, *n* (%)				0.81
Women	129 (87)	90	91	
Men	19 (13)	10	9	
Educational attainment, *n* (%)				0.014
<3 years higher education	57 (40)	42	26	
≥3 years higher education	85 (60)	56	73	
Missing, *n*	6	2	1	
Physical activity^a^, *n* (%)				0.001
Low	29 (23)	18 (21)	7 (8)	
Moderate	50 (40)	34 (41)	25 (27)	
High	47 (37)	32 (38)	59 (65)	
Missing, *n*	22	16	9	
Psychological well-being ≤ 50^b^, *n* (%)				<0.001
No	75 (51)	48	89	
Yes	73 (49)	52	11	
Missing, *n*	1	1		
Stress^c^, *n* (%)				< 0.001
Low	61 (42)	38	66	
Moderate	33 (22)	22	21	
High	53 (36)	39	13	
Missing, *n*	1	1		
Poor self-rated health^d^				< 0.001
No	58 (39)	38	96	
Yes	90 (61)	62	4	
Migraine days/month, median (q1, q3)	6 (4, 10)	6 (3, 10)	-	-
TTH days/month, median (q1, q3)	9 (4, 18)	9 (4, 15)	-	-
NP days/month, median (q1, q3)	11.5 (5, 30)	13 (5, 30)	-	-
Chronic migraine, n (%)	41 (28)	28	-	-
Chronic TTH, *n* (%)	21 (14)	17	-	-
Chronic NP, *n* (%)	54 (37)	49	-	-

Independent-samples t-test was used to test for differences in age; Chi-square test was used to test for difference in sex, educational attainment, physical activity, psychological well-being, stress and poor self-rated health

*M* migraine, *NP* neck pain, *n* numbers, *q* quartiles, *q1 and q3* refer to first and third quartiles, *SD* standard deviation, *TTH* tension-type headache

^a^Assessed using the International Physical Activity Questionnaire (IPAQ short form) low, moderate and high physical activity was based on time and energy expenditure in the past 7 days

^b^Assessed using the World Health Organisation five-item Well-Being Index (WHO-5) sum score from 0-100; sores ≤ 50 indicate poor psychological well-being

^c^Assessed using stress scores with end-points 0 = no stress at all and 10 = very high degree of stress; scores 0–3 = low, scores 4–6 = moderate, scores 7–10 = high stress

^d^Assessed using self-rated health with end-points 1 = very poor to 5 = very good; scores 1–3 indicated poor self-rated health

NP associated with the migraine attack was reported by 54%, NP associated with TTH was reported by 94% and NP without headache was reported by 66%.

Persons with migraine and co-existing TTH and NP were significantly less physically active, reported significantly lower psychological well-being, had higher levels of perceived stress and poorer self-rated health compared to healthy controls (Table 1).

Logistic regression analyses controlled for educational attainment showed that migraine and co-existing TTH and NP were strongly associated with physical activity, psychological well-being, perceived stress and poor self-rated health (Table 2).

**Table 2:** Odds ratios of co-existing M, TTH and NP and health-related variables controlled for educational attainment

	OR^a^ (95% CI)	*p* value	OR^b^ (95% CI)	*p* value
Physical activity^c^				
High	Ref		Ref	
Moderate	2.5 (1.28 – 4.91)	0.007	2.7 (1.35 – 5.36)	0.005
Low	4.7 (1.79 – 12.55)	0.002	4.4 (1.62 – 11.72)	0.004
Missing, *n*	16			
Psychological well-being ≤ 50^d^				
No	Ref		Ref	
Yes	8.8 (4.19 – 18.36)	<0.001	9.3 (9.35 – 19.86)	<0.001
Missing, *n*	1			
Stress^e^				
Low	Ref		Ref	
Moderate	1.8 (0.89 – 3.73)	0.103	1.6 (0.74 – 3.32)	0.238
High	5.2 (2.48 – 10.96)	<0.001	6.0 (2.78 – 12.98)	<0.001
Missing	1			
Poor self-rated health^f^				
No	Ref		Ref	
Yes	39.2 (13.32 – 115.15)	<0.001	37.7 (12.77 – 111.52)	<0.001

The analyses were conducted by binary logistic regression and estimated as OR and 95% CI

*CI* confidence interval, *M* migraine, *NP* neck pain, *OR* odds ratio, *Ref* reference value, *TTH* tension-type headache

^a^OR = unadjusted

^b^OR = adjusted for educational attainment

^c^Assessed using the International Physical Activity Questionnaire (IPAQ short form) low, moderate and high physical activity was based on time and energy expenditure in the past 7 days

^d^Assessed using the World Health Organisation five-item Well-Being Index (WHO-5) sum score from 0 to 100; sores ≤ 50 indicate poor psychological well-being

^e^Assessed using stress scores with end-points 0 = no stress at all and 10 = very high degree of stress; scores 0–3 = low, scores 4–6 = moderate, scores 7–10 = high stress

^f^Assessed using self-rated health with end-points 1 = very poor to 5 = very good; scores 1–3 indicated poor self-rated health

For persons with migraine and co-existing TTH and NP, the ability to perform physical activity was highly reduced because of migraine (median score of 9 on a rating scale from 0–10), moderately reduced because of TTH (median 5) and less reduced because of NP (median 3).

Almost half of the persons with migraine and co-existing TTH and NP (48%) rated migraine as the most burdensome of the three conditions. TTH was rated as the most burdensome condition by 30%, and NP was rated as the most burdensome condition by 10%. Migraine and TTH were rated as equally burdensome by 6%, all three conditions as equally burdensome by 3% and TTH and NP by 3%.

## Discussion

To the best of our knowledge, this is the first study to investigate the prevalence of migraine and co-existing TTH and NP in a clinical migraine population and to evaluate physical activity, psychological well-being, perceived stress and self-rated health in this subset of persons with migraine.

The prevalence of migraine with co-existing TTH and NP differed from the population-based study by Ashina et al. [3] that found a one-year prevalence of NP of 89.3% in persons suffering from migraine and co-existing TTH. The difference may be due to the sub-grouping of participants applied by Ashina et al. that investigated NP in persons with migraine and co-existing TTH. Molarius and Tegelberg [28] reported findings similar to ours although their case ascertainment was not based on ICHD-criteria.

In accordance with our study Varkey et al. [30] found that persons with migraine were less physically active compared to headache-free individuals and low physical activity was associated with a higher prevalence of migraine. Interestingly, in a population study, Ashina et al. [31] found that low physical activity was associated with migraine and co-existing TTH to a higher degree compared to other types of headache and the association was stronger with TTH than migraine only. Physical activity may be a potential migraine treatment strategy to reduce migraine frequency, pain intensity and duration [6]. Both physical activity and stress reduction strategies have been suggested to increase the quality of life [32–34]. Moreover, systematic stress management, such as progressive muscle relaxation, reduced migraine frequency [7] and a relaxation program has been shown to be effective in persons with headache and neck/shoulder pain [12].

The characteristics of the participants with migraine and co-existing TTH and NP reflect the findings of other studies on the impact of migraine on psychological well-being, an association with increased stress and poor self-rated health [28, 35–37]. Schramm et al. [37] did not find any differences in the level of perceived stress between those with migraine only and those with migraine and co-existing TTH. Molarius and Tegelberg [28] found that persons with migraine and recurrent headache reported poorer self-rated health than those with migraine only. Reduced psychological well-being may indicated stress or depression [23]. Persons with migraine are in general very susceptible to stress [38, 39] and have higher levels of stress compared to headache-free individuals [37] which is in line with our findings.

This study showed that persons with migraine and co-existing TTH and NP had very low level of physical activity, and their perceived ability to perform physical activity was reduced not only owing to migraine (to a high degree) but also TTH and NP (to a lesser degree), although almost 50% suffered from chronic NP. In contrast to our study, the population study by Ashina et al. [31] found a higher association between low physical activity and TTH only, than between low physical activity and migraine only. Compared to the population study, we recruited persons with migraine which can explain these different results.

Our results suggest that persons with migraine and co-existing TTH and NP may have more barriers to perform physical activity compared to those with migraine only, even though TTH and NP were not rated as quite as burdensome. Decreased psychological well-being, high perceived stress and low self-rated health among those with migraine and co-existing TTH and NP suggest conditions that may make it more difficult to motivate this group to increase their levels of physical activity.

Migraine, TTH and NP are complex and multi-factorial disorders. It is important to consider implementing multidisciplinary treatment strategies when these three conditions are co-existing [40, 41]. Whether a physiotherapeutic treatment modality such as physical activity has a mutual or complementary effect on migraine and co-existing TTH and NP has yet to be investigated.

Strengths of this study were that the participants’ diagnoses were based on a 4-week diagnostic headache diary before their first visit in the headache centre together with a detailed headache history and a neurological examination. Also, an additional interview was conducted to ascertain characteristics of migraine, TTH and NP. This procedure reduced the risk of misclassification and excluded other neurological diseases. The ICHD 3-beta criteria for migraine and TTH were used, and NP was described using the classification system by the Neck Pain Task Force [19]. Also, all participants were presented with a drawing of the anatomic region of the neck in order to standardise descriptions of NP distribution area.

The response rate of persons with migraine with returned questionnaire was relatively high [3, 28] making our estimate of the one-year prevalence of migraine with co-existing TTH and NP relatively strong.

We used three different variables (psychological well-being, perceived stress and self-rated health) as measures of health-related quality of life. These represent different domains and together give a good indication of the headache burden and what is important to consider when deciding a relevant treatment strategy and/or outcome measures for migraine and co-existing TTH and NP.

Limitations of the study were that participants were recruited from one tertiary referral headache centre and may, therefore, not represent the total migraine population. The participants had a relatively high prevalence of both chronic migraine (28%) and chronic TTH (17%), which may explain our large proportion of persons with migraine and co-existing TTH and NP. Further, such a clinical sample may be more aware of their symptoms and more capable of distinguishing between migraine and TTH than the general migraine population.

Most of the healthy controls were recruited among healthcare professionals, and were generally more highly educated than the persons with migraine and co-existing TTH and NP and they were more physically active than the persons with migraine and co-existing TTH and NP and the general population [42]. However, they were very close to the general population regarding sum scores of psychological well-being [43]. Education was therefore considered as a confounding factor in the regression analyses.

Self-report on physical activity, psychological well-being, stress and self-rated health may increase the risk of recall-bias. Personal interview is stronger than self-report in reducing recall bias, and might have prevented the exclusion of participants who had inadequate answers in the IPAQ questionnaire. The drawback is that interview may cause interviewer bias. Prospective diary recording of migraine and co-existing TTH and NP together with personal interview may have been more ideal as diagnostic tools [44].

Detailed history and diary recording are particularly important for ascertaining multiple diagnoses. In the future, a larger sample of participants would allow for separate analyses of those with migraine only and migraine with either co-existing TTH or NP. This would also allow controlling for migraine disability.

There is a risk of losing power when collapsing responses into few categories, however, these categories are more informative and easier to explain to patients.

## Conclusions

Migraine and co-existing TTH and NP was highly prevalent in a clinical sample of persons with migraine. Persons with migraine and co-existing TTH and NP reported significantly lower level of physical activity and psychological well-being; higher level of perceived stress, and poorer self-rated health than healthy controls. Persons with migraine and co-existing TTH and NP reported a reduced ability to perform physical activity owing to all three conditions with migraine as the most burdensome condition followed by TTH and NP. Persons with migraine and co-existing TTH and NP may require more focused interventions to increase physical activity. Whether physical activity is a beneficial treatment modality for this group is not known, and should be investigated in a clinical trial.

## Abbreviations

CI: Confidence interval
ICHD: International classification of headache disorders
IPAQ: International physical activity questionnaire
MET: Metabolic equivalent task
NP: Neck pain
OR: Odds ratio
TTH: Tension-type headache
WHO-5: The World Health Organization 5-item Well-Being Index
